# Systems Biology – A Guide for Understanding and Developing Improved Strains of Lactic Acid Bacteria

**DOI:** 10.3389/fmicb.2019.00876

**Published:** 2019-04-30

**Authors:** Jianming Liu, Siu Hung Joshua Chan, Jun Chen, Christian Solem, Peter Ruhdal Jensen

**Affiliations:** ^1^National Food Institute, Technical University of Denmark, Kongens Lyngby, Denmark; ^2^Carl R. Woese Institute for Genomic Biology, University of Illinois at Urbana–Champaign, Champaign, IL, United States; ^3^Department of Chemical and Biological Engineering, Colorado State University, Fort Collins, CO, United States

**Keywords:** food fermentation, metabolic engineering, strain development, control analysis, screening and selection

## Abstract

Lactic Acid Bacteria (LAB) are extensively employed in the production of various fermented foods, due to their safe status, ability to affect texture and flavor and finally due to the beneficial effect they have on shelf-life. More recently, LAB have also gained interest as production hosts for various useful compounds, particularly compounds with sensitive applications, such as food ingredients and therapeutics. As for all industrial microorganisms, it is important to have a good understanding of the physiology and metabolism of LAB in order to fully exploit their potential, and for this purpose, many systems biology approaches are available. Systems metabolic engineering, an approach that combines optimization of metabolic enzymes/pathways at the systems level, synthetic biology as well as *in silico* model simulation, has been used to build microbial cell factories for production of biofuels, food ingredients and biochemicals. When developing LAB for use in foods, genetic engineering is in general not an accepted approach. An alternative is to screen mutant libraries for candidates with desirable traits using high-throughput screening technologies or to use adaptive laboratory evolution to select for mutants with special properties. In both cases, by using omics data and data-driven technologies to scrutinize these, it is possible to find the underlying cause for the desired attributes of such mutants. This review aims to describe how systems biology tools can be used for obtaining both engineered as well as non-engineered LAB with novel and desired properties.

## Introduction

Lactic acid bacteria (LAB) are traditionally used as starters in fermented food production. LAB produce lactic acid, the presence of which reduces growth of pathogens and other undesirable microbes in the products, while contributing to an appealing acidic taste. LAB are also capable of producing various aromatic compounds, the formation of which are initiated by lipolysis and proteolysis, and these also contribute to the pleasant organoleptic characteristics of different fermented products such as yogurt, cheese, and butter (Song et al., 2017). LAB are thus highly industrially relevant microorganisms and represent a multi-billion dollars business worldwide (Johansen, 2017).

Several important phenotypical characteristics of LAB such as acidification rate (Martinussen et al., 2013), robustness to environmental stresses and phage resistance (Garneau and Moineau, 2011; Papadimitriou et al., 2016), contribution to flavor and texture formation (Broadbent et al., 2005; Chen J. et al., 2017), bio-protection activity (Siedler et al., 2019) and probiotic function (Ljungh and Wadström, 2006), are important for real-life applications and are continuously being investigated by both the academic and industrial community. Traditional non-GMO (genetically modified organism) strain modification approaches such as random mutagenesis and screening, selection using toxic analogs and adaptive evolution, have a long history of successful use for improving properties of LAB. The use of mutagenesis for improving LAB is only constrained by the genetic repertoire of the bacteria, but a large screening input is usually required to identify desired mutants. The latter two methods are specific and very selective, but their implementation is largely condition-dependent. For analog selection, the target pathway should be tightly associated with anabolism, and an effective metabolite analog should also be easily accessible. For adaptive evolution, the selection is dependent on fitness improvements under stressful conditions. Rational strain modification methods, such as metabolic engineering and synthetic biology, have demonstrated their effectiveness to endow LAB with new/improved characteristics useful for food applications beyond what traditional approaches are capable of delivering. However, since LAB are often present in the final product destined for human consumption, there are still hurdles that have to be overcome before recombinant DNA technologies can be widely implemented, e.g., regulatory issues and skepticism of consumers.

The understanding of LAB physiology has fundamentally changed since the emergence of high-throughput genome sequencing technologies. By using whole-genome sequencing to compare the genome sequences of model LAB strains and their derivatives generated using traditional strain improvement procedures, researchers have gained great insight into how phenotypes are affected by genetic variations. Modern sequencing technologies allow for entire genome landscapes of multiple bacteria to be generated simultaneously within less than 24 h. These genome sequence data can be used as references, when doing physiological characterization or when using various systems biology approaches to address different fundamental and practical questions.

Due to their Generally Recognized as Safe (GRAS) status, simple metabolism, and a very high glycolytic flux (Koebmann et al., 2002a), LAB recently have emerged as promising cell factories for production of high-value biochemicals, including food ingredients and pharmaceutical precursors. This has been aided by genome-scale metabolic models, where researchers have used *in silico* simulations to find the best way to reroute LAB metabolic networks to optimize production of various compounds. In this review we will discuss four main approaches used for improving LAB: (1) Adaptive Laboratory evolution (ALE) in combination with meta-omics analysis for characterization of mutants; (2) Systems biology tools for elucidating microbial interactions and metabolic capacities; (3) a detailed analysis on metabolic flux regulation for LAB model strain – *Lactococcus lactis*; (4) metabolic engineering of *L. lactis* as a novel microbial cell factory.

## Lab and Adaptive Laboratory Evolution

### Bacterial Evolution Is a Consequence of Inherent Genetic Variations and Natural Selection

In contrast to the comparative genomics approach for studying the inter/intraspecies relationship of LAB, ALE studies focus on genomic adaptation, including single nucleotide variations (SNV), deletions and insertions, to specific environmental stresses or metabolic perturbations, and its correlation to phenotypical changes, where the latter are typically studied using a combination of omics analysis, e.g., transcriptomics, proteomics, metabolomics, and physiological characterization. In an ALE setup, the number of accumulated mutations is normally controlled by limiting the number of generations of propagation in a subculturing or chemostat system. In contrast to natural evolution, the type and dose of selection stress, which serve as an input variable, can easily be defined in a laboratory environment. This approach puts a limit on the complexity of the subsequent comparative systems biology study, by limiting the number of output variations. The eventual multi-omics analysis and data integration with systems biology modeling are expected to provide the foundation for a better understanding of the evolutionary driving force of LAB, and to guide the rational design of ALE conditions to improve the performance of industrial LAB (Bachmann et al., 2017).

Adaptive laboratory evolution has been extensively used in stress physiological studies of LAB. As LAB are industrially important workhorses, LAB often undergo a variety of environmental stresses e.g., heat, salt and acid stress in different manufacturing conditions. Heat is one of the common stresses that LAB need to handle well in industrial processes. For instance, the mesophilic *L. lactis*, the main constituent in starters used for making semi-hard cheeses, such as cheddar and Gouda, is exposed to high temperatures during the syneresis step where the moisture content in the cheese grains is reduced. Such suboptimal temperatures are not lethal to *L. lactis*, but the physiological properties of *L. lactis* are significantly altered to manage the harsh conditions. LAB normally display a multi-level cellular response to environmental stresses, where multi-omics serves as desirable analysis tools for understanding the mechanism. To study the high-temperature physiology and its molecular basis, Chen et al. (2015a) applied ALE on the model *L. lactis* strain MG1363 with gradually increased incubation temperature. After an 800-generation session of experimental adaptation at high temperatures using a serial-transfer regime, a thermal-tolerant variant was isolated. The authors characterized the mutant with comprehensive systems biology analysis. On the metabolic level, the mutant had expanded its glycolysis capacity at high temperatures, where the overall glycolytic flux in the mutant was 13% higher compared to that of the wild-type strain at 38°C. On the transcriptomic level, a variety of solute transport activities had been enhanced in the mutant including ones for carbohydrates, amino acids and ions. Alterations in cell-membrane fatty acid composition were also observed for the mutant, which had an increased amount of saturated fatty acids (C14:0 and C16:0) in the cell membrane of the thermally tolerant mutants. Increased amounts of saturated fatty acids help maintain the fluidity of cell membranes at high temperatures and thereby stabilizing the cross-membrane transport activities (Los and Murata, 2000). On the genomic level, the authors identified 13 mutations through whole genome resequencing, and subsequently used reverse engineering and physiological characterization to determine the contribution of individual mutations to the overall phenotype. It was found that SNVs in *groESL* (encoding chaperon proteins) and *rpoC* (encoding RNA polymerase subunit) contributed significantly to thermal tolerance of the mutant. The SNVs in *groESL* led to overexpression of molecular chaperones, and the SNVs in *rpoC* caused the alteration in global gene expression, and as a consequence of this, the saturated fatty acid synthesis pathway was overexpressed. The integrated multifaceted analysis indicated that a stable cell membrane structure in combination with other events, such as overexpression of molecular chaperones, is important for the cells to support a high-energy turnover rate under heat stress conditions. The study demonstrates how multi-omics analysis can help understand the stress physiology of LAB on the molecular level.

Besides the environmental stresses, LAB such as *L. lactis* often encounter nutrient starvation conditions during cheese ripening. In the early stage of ripening, acidification continues with limited lactose (3–5% lactose of milk depending on moisture) in the curd after whey drainage (Ardö and Nielsen, 2007). After the carbohydrate is completely exhausted, the cells stop growing or eventually lyse (Ganesan et al., 2007). The global catabolite regulator CcpA plays an important role in the regulation of the metabolic shift under these carbohydrate limitation/starvation conditions, but how LAB reshape the cellular metabolism has still not been elucidated. In contrast to traditional ALE with the serial-passage regime, the growth rate can be controlled by limiting specific nutrients in a chemostat ALE setup, which is used to mimic the slow growth and metabolic activity of LAB during cheese ripening (Van Mastrigt et al., 2018). Price et al. (2017) performed a glucose-limited ALE for *L. lactis* in a chemostat setup to study the response to carbohydrate starvation. After a few generations of adaption, fast-growing *L. lactis* mutants emerged and finally dominated the population. Whole genome resequencing of the isolates obtained from parallel evolution lineages indicated that the SNVs causing a site-specific change in the amino acid sequence in CcpA (Met-19) was responsible for the adaptation. To study the global regulatory role of the mutated CcpA, they performed a transcriptomics analysis, and found that glucose transport in the mutant had changed. The mutation in CcpA led to an overexpression of PTS^Man^ but a downregulation of PTS^Cel^. Both PTS^Man^ and PTS^Cel^ participate in the glucose transport in *L. lactis*, but PTS^Man^ has a higher substrate affinity for glucose compared to PTS^Cel^ (Castro et al., 2009). The CcpA-mediated transcriptional change caused a 3-fold increased rate in glucose uptake in the mutant, which was further corroborated by the flux analysis with C^14^-glucose. These findings suggest that there is an evolutionary advantage associated with altering global regulators when compared to altering expression of individual genes, at least in response to carbohydrate starvation (Goel et al., 2015).

### LAB and Genome-Scale Metabolic Modeling

Since the first LAB genome sequence (*L. lactis* subsp. *lactis* IL1403) was announced in 2001 (Bolotin et al., 2001), whole genome sequencing has significantly accelerated systems biology research on LAB. Based on the genome annotation, Genome-Scale Metabolic models (GEMs) with Constraint-Based Reconstruction and Analysis (COBRA) have been used to predict the nutrient requirements for growth, and metabolic patterns for LAB under different conditions (Wu et al., 2017). This information is paramount for both starter culture producers as well as for the food industries relying on LAB. The accuracy of metabolic models depends on correct genome annotations, and subsequent manual curation with biochemical, genetic and cell physiological data (Kitano, 2002a). Several studies have suggested that the NADH/NAD^+^ ratio, which depends on the sugar uptake rate, has a role in the shift between the homolactic and mixed-acid fermentation in LAB (Garrigues et al., 1997), and that LAB with a low glycolytic flux need more energy to produce biomass. During mixed-acid fermentation, one extra ATP is generated when acetate is formed from acetyl-CoA. The experimental observation that biomass yield is maximized under starvation conditions has also been predicted using GEMs (Oliveira et al., 2005). In another case, GEMs and COBRA were used to predict the outcome of an ALE, where *Lactobacillus plantarum* was grown on the non-conventional substrate glycerol. The question addressed was how the metabolic network could be reshaped to support fast growth. *L. plantarum* grows slowly on glycerol, but only under aerobic conditions. Teusink et al. (2009) adapted *L. plantarum* on glycerol using a complex medium. After approximately 800 generations, fast-growing mutants could be isolated. Metabolic flux analysis showed that lactate rather than acetate was the major product from glycerol dissimilation. It was assumed that a mixed-acid fermentation mode was needed to support a high-energy yield on slowly fermentable sugars. In this case, the specific growth rate of the mutant (0.26 h^-1^) on glycerol was still substantially lower than the growth rate on other fast-fermentable sugars. To explain the observations, the authors applied flux balance analysis (FBA) using the uptake rates of glycerol, citrate (already present in the medium) and oxygen as constraints. As the flux model was set to maximize the biomass yield under aerobic conditions, the oxygen consumption played an important role in FBA. Under aerobic conditions, the model predicted that more ATP could be generated when lactate was formed, and that lactate production would support faster growth. The good correlation between the model prediction and experimental outcome demonstrates that COBRA is a valuable tool for predicting how the metabolic network of LAB adjusts over the course of an evolution experiment.

### ALE and Genomics Analysis

Adaptive laboratory evolution in combination with genomic analysis can also help disclose fitness-associated gene functions in LAB. LAB such as *Lactobacillus* and *Lactococcus* species, are facultative anaerobes. Albeit the lack of respiration, the presence of oxygen usually does not affect normal growth (Higuchi et al., 2000). The toxicity of oxygen toward LAB is believed to be due to generation of reactive oxygen species (ROSs) during oxygen metabolism (Chen et al., 2013). Due to their catalase negative nature, LAB are more vulnerable to the attack from H_2_O_2_-derived ROSs. Oxygen not only affects the cell fitness, but also tends to alter the acidification profile of LAB cultures (Larsen et al., 2015). Therefore, the study of oxygen related gene functions in LAB has high industrial relevance. The thioredoxin-thioredoxin reductase is the major system for maintaining the cellular redox homeostasis of LAB when oxidative stress is imposed (Vido et al., 2005; Serrano et al., 2007; Serata et al., 2012). Two thioredoxin reductase genes are annotated on the chromosome of *L. lactis* namely *trxB1* and *trxB2*. The importance of *trxB1* for regulating redox homeostasis has been demonstrated by single gene knockout experiments in *L. lactis* MG1363. The loss of functional TrxB1, however, is not lethal for *L. lactis* in the presence of oxygen (Vido et al., 2005). Chen et al. (2015b) introduced a deletion in *trxB2*, and noticed that aerobic growth of the mutant was substantially retarded. TrxB2 shares a high amino acid sequence similarity with TrxB1, but it lacks two conserved cysteine residues, which are necessary to function as a thioredoxin reductase. To elucidate the biological function of TrxB2 under aerobic conditions, the authors designed an ALE setup, where the mutant was subcultured under aerobic conditions for a prolonged period of time. Genome resequencing of adapted mutants revealed the occurrence of suppressor mutations in the ribonucleotide reduction pathways in mutants isolated from independent lineages. The research suggested an important role of TrxB2 as a flavodoxin reductase in the aerobic ribonucleotide reduction, which was supported by subsequent physiological characterization (Chen et al., 2015b). Often, the physiology studies of LAB under oxidative stress conditions focus on ROS, as ROS has detrimental effects on cell fitness (Guchte et al., 2002). However, oxygen-dependent anabolism should not be underestimated in terms of its importance for aerobic growth of LAB.

### ALE and Multiomics Analysis

Adaptive laboratory evolution and multiomics analysis have been demonstrated to be useful tools for addressing fundamental questions in the natural evolution of LAB. The comparative genomics study of different *L. lactis* isolates suggests that the dairy-associated *L. lactis* diverged from the plant isolates through a long history of natural evolution (Siezen et al., 2008). The phenotypic properties are quite different between the non-dairy (plant, meat and bovine rumen) isolates and dairy-associated isolates in terms of nutrient requirements and stress tolerance in different environments. Some unique traits e.g., excellent flavor formation capacity from amino acid catabolism of the plant-associated *L. lactis* strains has also been used for enhancing the flavor formation in dairy fermentation (Tanous et al., 2005). However, the important question of gene gain and loss during the natural evolution of the plant isolates adapting the dairy niche has not been fully addressed. Bachmann et al. (2012) adapted the plant-derived *L. lactis* strain KF147 in milk for nearly 1,000 generations. Isolates obtained from three independent evolutionary lineages had adapted to growing well in milk. The integrated comparative genomics and transcriptomics analysis revealed that the adapted strains had variations that resulted in improved utilization of milk proteins and loss or down-regulation of pathways important for using plant materials. Such gene gain and loss are a typical feature found in the natural evolution of LAB accommodated by a dairy niche (Makarova and Koonin, 2007; Hanemaaijer et al., 2015).

## From Single-Strain to Community-Based Systems Biology

### Metagenomics

It is a common practice that mixed starter cultures (the combination of different strains) are used for food fermentations, e.g., for production of cheese and yogurt. One consideration is that the process with a single strain is more vulnerable to bacteriophage predation, where strain-specific phages can result in fermentation failure. This problem is alleviated when mixed strains are used, as strains unaffected by these phages will ensure that the fermentation does not fail (Smid et al., 2014).

The use of mixed strains also confers the culture a broader metabolic capacity, which is important to achieve a desirable flavor and organoleptic property of the final product. In particular for cheeses made using mesophilic starters cultures this is relevant. The mesophilic starter cultures are typically composed of *L. lactis* subsp. *cremoris, L. lactis* subsp. *lactis* and its citrate-positive variant (*L. lactis* subsp. *biovar diacetylactis*). During milk fermentation, both *L. lactis* subsp. *cremoris* and *L. lactis* subsp. *lactis* contribute to acidification. During the subsequent cheese ripening stage, the roles of different strains for flavor formation become distinct (Ardö and Nielsen, 2007).

Mixed strains can be rationally designed by blending two or more well-characterized industrial strains on the condition that their growth dependency and product profiles meet the quality requirement such as the mesophilic/thermophilic cultures used in cheese/yogurt manufacturing (Sieuwerts, 2016). On the other hand, mesophilic undefined starter cultures are commonly used for the manufacture of the European continental semi-hard cheeses such as Cheddar, Gouda, and Danbo. Such cultures derive from back-slopping cultures, which were often collected from the batches yielding good quality cheese from artisanal cheese operations, and saved at –80°C to minimize changes in LAB composition during storage (Smid et al., 2014). Such undefined starters have a long history of successful use for manufacturing cheeses with a rich flavor, and are highly phage resistant, most likely due to the coevolution of the strains and exchange of phage resistance mechanisms.

To study the undefined starter culture, it is necessary first to characterize starter composition, i.e., determine the strains it contains and the corresponding amounts, before characterizing single-strain physiology and genetic background (Temmerman et al., 2004). Culture dependent approaches are commonly used, however, are tedious and the empirical choice of medium for enumeration does not ensure the recovery of the entire community of strains (Erkus et al., 2013; Frantzen et al., 2016). Culture-independent approaches for characterization scarcely provide detailed information about community dynamics and interaction in practice. It is desirable to transfer systems biology strategies used for characterizing single strains to mixed strains, but an understanding of community-level genomics is necessary before systems biology approaches can be applied to study microbial consortia.

Metagenomics analysis provides insights into the species composition and its dynamics in a culture-independent manner. The holistic decoding of the genomic material in an undefined starter community via metagenomics studies definitely benefits the research of starter LAB e.g., high-resolution surveillance of the microbial community dynamics or construction of community-based GEMs. Such knowledge will facilitate the general understanding of growth, metabolism and physiology during mixed culture preparation, cheese manufacturing and ripening (Jonnala et al., 2018). Use of 16S amplicon sequencing and shotgun metagenomics are typical in metagenomics studies of microbial consortia. Sequencing amplicons of conserved regions in 16S rRNA using Next-Generation Sequencing (NGS) technologies provide information about species-level community composition on how composition changes in response to different abiotic factors (De Filippis et al., 2017). There are two limitations, which should be considered when using 16S amplicon sequencing to determine composition. First, the variation in 16S rRNA allele numbers in LAB prevents a precise prediction of abundance, if the copy number is not known for individual strains. Second, 16S amplicon sequencing only enables the species-level differentiation. Especially for undefined mesophilic starter cultures, the resolution of 16S amplicon sequencing is low, as it is mainly composed of the *L. lactis* species with a low 16S rRNA sequence variation. For example, Porcellato and Skeie (2016) accessed the impact of elevated cooking temperature on cheese microbiota composition. The authors used 16S amplicon sequencing and found that a high temperature led to a reduced number of live *L. lactis* cells during ripening. *L. lactis* subsp. *lactis* is generally more stress tolerant compared to *L. lactis* subsp. *cremoris*, but the 16S amplicon sequencing could not deliver the subspecies information of the inactivated *L. lactis* in this particular study. Furthermore, 16S amplicon sequencing only provides information about rRNA sequences, but hardly provides deep insight into the metagenome.

**FIGURE 1:**
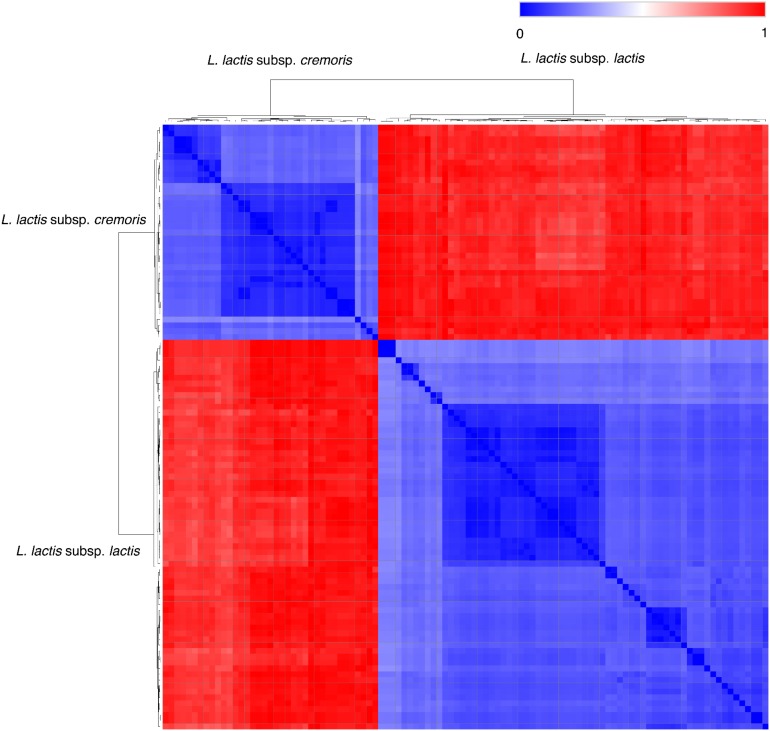
Hierarchical clustering of 103 published lactococcal genomes. The genome sequencing data were obtained from NCBI. The distance of phylogeny was calculated using MASH. The distance data generated by MASH (Ondov et al., 2016) was used to performing the hierarchical clustering on Morpheus available from the Broad Institute (https://software.broadinstitute.org/morpheus/).

With improved quality and decreased price of second-generation shotgun sequencing, it is now possible to do deep-sequencing metagenomics and determine the entire genomic content (core/pan-genome) of a microbial ecosystem, including the low-abundant strains. Using this approach, genome decay (plasmid-loss) and the species-level dynamic shift of the community due to the biotic/abiotic factors can be revealed while comparing the reads to marker databases for abundance calculation (De Filippis et al., 2017). Nevertheless, there are only few published studies where metagenomics analysis has been used to study starter culture composition at the strain-level. The high coverage sequencing eases the identification of metabolic genes in the strain community, but the challenges remains as to which strains the genes belong. One technical shortcoming of second-generation sequencing platforms is that only short reads (<800 bp) are generated, which prevents the strain-level differentiation with the *de novo*-based assembly based metagenomics analysis. Especially in the mesophilic-undefined starters, the dominating *L. lactis* species exhibits a high intra-subspecies genome similarity (Figure 1). Therefore, the typical contig binning steps by GC content, coverage or metabolic networks are difficult to apply on the strain-level assembly (Albertsen et al., 2013; Biggs and Papin, 2015).

Recently, several new data analysis tools based on reference-mapping approaches have been developed and validated which can be used for strain-level metagenomics data analysis (Scholz et al., 2016; Albanese and Donati, 2017; Truong et al., 2017; Zolfo et al., 2017). The emergence of these new tools has attracted the attention from the LAB scientific community as it now is possible to achieve a more profound understanding of the starter culture community (structure and function) and its contribution to the quality of fermented products (Ercolini, 2017). The development of the third-generation long-read sequencing technology is booming. Researchers have demonstrated single-read sequencing up to 0.9 Mbp length using the MinION system (Jain et al., 2018). Considering the comparable small genome size of LAB (1.2–4.9 Mbp) (Douillard and de Vos, 2014), in the future, with further improvements of throughput and accuracy, the third-generation genome sequencing platforms with long-read sequencing ability will definitely facilitate *de novo* assembly of single-strain genomes in undefined starter cultures, and eventually facilitate system-level understanding of the community (Turaev and Rattei, 2016). Another promising approach for looking at single-cell genomics in microbial communities is to use single-cell sequencing technologies. The concept is to first sort and compartmentalize single cells in water-in-oil droplets, where the DNA is isolated and barcoded in an isolated environment prior to whole genome amplification of single chromosomes followed by sequencing (Gawad et al., 2016). The main challenge for this technology is the resolution. If the sequencing accuracy can be improved in the future, it can largely facilitate the strain-level genomics study of microbial consortia.

### Postgenomic Studies of LAB

Genomics data are the basis of systems biology (Kitano, 2002b). Postgenomic analysis tools such as transcriptomics, proteomics and metabolomics have also been widely used to understand the physiology of LAB at a systems level. A large number of these kinds of analyses have been applied to single strains and they provide great insights into how cells respond to different abiotic/biotic perturbations. To thoroughly understand industrially relevant properties of LAB, useful omics data should be generated from the LAB consortia in action, i.e., in dairy fermentations that are similar to those taking place in dairy plants, and there are only few such studies. The yogurt culture is a well-described mutualistic system, in which defined *Streptococcus thermophilus* (*S. thermophilus*) and *Lactobacillus delbrueckii subsp*. *bulgaricus* (*L. bulgaricus*) strains are mixed for co-culture fermentation of bovine milk. In the mutualistic life of the yogurt culture, the growth profiles of *S. thermophilus* and *L. bulgaricus* show a clear protocooperation relationship (Sieuwerts, 2016). As the LAB blended in the yogurt starter culture are normally well-documented, it serves as a simplified model system for the implementation of system biology study for microbial community. The balanced growth of these two bacteria is important for fast acidification and desirable texture and flavor formation of yogurt (Chen C. et al., 2017; Yamauchi et al., 2019). In yogurt fermentations, both the growth and acidification are stimulated when cocultures of the two LAB are used. It appears that the protocooperation occurs in the manner of nutrient exchange, where production of formic acid and carbon dioxide from *S. thermopilus* stimulates the growth of *L. bulgaricus*, and the proteolytic activity of *L. bulgaricus* provides essential peptides and amino acids to *S. thermophilus.* The integrated transcriptomics and proteomics analysis of *S. thermophilus* in coculture with *L. bulgaricus* confirmed the mutualistic effects, where upregulation of anabolic pathways in both *S. thermophilus* and *L. bulgaricus* compared to monoculture cultivation was observed (Herve-Jimenez et al., 2009; Sieuwerts et al., 2010). Meta-transcriptomics and meta-proteomics analyses revealed a dynamic response to both biotic and abiotic factors at different stages of the mutualistic growth, results that largely confirm the previously observed phenotypic behavior between the cooperation of these two bacteria. Interestingly, ion homeostasis was affected in *S. thermophilus* in the coculture with *L. bulgaricus* (Herve-Jimenez et al., 2009; Sieuwerts et al., 2010). Reduced ion transport and increased ion-chelating activity in *S. thermophilus* was shown to be correlated with H_2_O_2_ production by *L. bulgaricus*. The major oxidative stress reponse system, however, was not induced in *S. thermophilus*. One reason for this was that *L. bulgaricus* produces minor amounts of H_2_O_2_ during growth in milk. The adaptation of the catalase-negative *S. thermophilus* to H_2_O_2_ by control of the intracellular ion homeostasis gives an indirect way to circumvent the oxidative stress.

The cheese environment is a more complex ecosystem than yogurt and also the cultures used are more complex. Typically, different kinds of LAB e.g., *lactococci* and *streptococci* are involved in acidification and cheese ripening. Cheese ripening is one of the most important processes in cheese manufacturing in terms of flavor formation. However, ripening is a slow process. Not only starter LAB but also other non-starter LAB (NSLAB) usually have an important contribution to flavor formation during cheese ripening. To accelerate cheese maturation, one option is to elevate the ripening temperature to increase the rate of the biochemical transformations taking place. Meta-transcriptomics (RNA-seq) data shows that expression of genes involved in proteolysis, lipolysis and amino acids/fatty acids catabolism are promoted in NSLAB at higher ripening temperatures, and in turn, an accelerated maturation has been noticed (De Filippis et al., 2016).

Besides meta-transcriptomics, meta-proteomics and metabolomics are also important tools for studying LAB. Currently only very few works have used these two technologies to study LAB communities. Reasons could be due to the challenges in sample preparation, proteins and metabolites identification compared to sequence-based metagenomics and meta-transcriptomics data in the community study (Blackburn and Martens, 2016; Smirnov et al., 2016).

The aforementioned ALE and metabolomics tools have been harnessed to obtain LAB that exhibit improved properties. We have also discussed the application of these systems biology tools to elucidate interactions taking place in LAB communities and to determine metabolic capacities. In the following part, we will focus on the model strain of LAB – *L. lactis* to elaborate its metabolic flux regulation in more detail and illustrate its metabolic engineering potential for valuable biochemicals production as a novel microbial cell factory.

## Metabolic Flux Regulation of *L. lactis* With a Focus on Glycolysis

The glycolysis of *L. lactis* comprises the typical EMP pathway with different carbohydrates entering at different points. Sugars enter cells of *L. lactis* by either the PEP-dependent PTSs or sugar-specific permeases. For glucose uptake, there are two distinct PTSs, PTS^Man^ and PTS^Cel^, and a proton-motive force dependent permease and the kinetic properties of these transport systems in MG1363 have been characterized (Castro et al., 2009). Glycolytic enzymes are in general highly expressed to sustain the large glycolytic flux in *L. lactis*, accounting for about 20% of the total soluble protein (Puri, 2014). Their genes are in general located close to the origin of replication for higher expression (Cocaign-Bousquet et al., 2002). Transcriptional regulation of the glycolytic genes in *L. lactis* primarily is through the carbon catabolite repression (CCR), which has been verified experimentally in *L. lactis* (Deutscher et al., 2006). The HPr protein, at high levels of fructose 1,6-bisphosphate (FBP) and ATP, is phosphorylated and then forms a complex with CcpA acting as a global regulator binding to the *cre* sites on chromosome. The well-conserved consensus in Gram-positive bacteria for the *cre* site is TGNNANCGNTNNCA, which is roughly palindromic with the central CG base always present. A study on the CcpA regulon in MG1363 proposed the consensus WGWAARCGYTWWMA, which is specific for *L. lactis* (Zomer et al., 2007). The binding of CcpA to *cre* sites can lead to either activation or repression, depending on the position relative to promoters also seen for *Bacillus* (Henkin, 1996). When a *cre* site is upstream of, inside, or downstream of the promoter of a gene, transcription is activated, repressed, or aborted accordingly (Cocaign-Bousquet et al., 2002). Results also have indicated that the interaction between CcpA and the transcription machinery may be dependent on the helix side of CcpA binding, because the strongest repression was observed for cre sites that were consecutively separated by around 10.5 bp, equal to a full helical turn of double-stranded DNA (Zomer et al., 2007). The expression of a number of glycolytic enzymes was found to be up-regulated by CcpA, including phosphofructokinase (PFK), pyruvate kinase (PYK) and lactate dehydrogenase (LDH) (the members of the las operon) (Luesink et al., 1998; Kok et al., 2017), phosphoglucose isomerase (PGI), glyceraldehyde 3-phosphate dehydrogenase (GAPDH), enolase (Guédon et al., 2002).

In the intracellular environment, assuming constant environmental factors, such as pH, temperature, viscosity etc., the reaction rate of an enzyme depends on concentrations of the enzyme, substrates, products and other effectors, such as allosteric regulators, that can modulate the enzymatic activity. Reaction rates would in turn change metabolite concentrations, forming a dynamical system. The metabolic flux of a pathway is the overall conversion rate of metabolites by the pathway resulting from the dynamical interactions between involved enzymes and metabolites. The factors determining the flux can be divided into two levels. The first is the enzyme level, which accounts for changes in fluxes caused by changes in gene expression level. The second is the metabolic level referring to changes in fluxes which are not caused by altered gene expression but by changes in metabolite concentrations and the inherent kinetic properties of enzymes such as maximum velocity and substrate affinity. The statement that an enzyme has “control” on a flux should refer to the phenomenon that a change in the enzyme level leads to change in the flux but not the direct regulatory mechanism. “Regulation” should refer to the exact mechanism causing the change (Chan, 2014).

### Control of the Glycolytic Flux by Individual Enzyme Levels

Control of fluxes by individual enzymes can be quantified by Flux Control Coefficients (FCCs) in the theory of Metabolic Control Analysis (MCA), which is defined by the rate of fractional change of the steady-state flux with respect to the fractional change of the enzyme activity. Finding the “rate-limiting” step or an enzyme with high FCC of the glycolytic flux in *L. lactis* can have direct industrial relevance, e.g., speeding up the production properly and increasing the productivity. An earlier study trying to inhibit the activity of GAPDH by the specific inhibitor iodoacetate indicated that GAPDH had a high FCC of about 0.9 on glycolytic flux in non-growing cells of *L. lactis* subsp. cremoris Wg2 (Poolman et al., 1987). Similar results were obtained in another strain NCDO2118 with GAPDH having a FCC equal to 0.7 (Even et al., 1999). Later, the control of glycolytic flux by glycolytic enzymes has been extensively studied by the Jensen group by experimental estimation of FCCs in the laboratory strains *L. lactis* IL1403 and MG1363. The results are summarized in Table 1.

**Table 1:** Summary of experimentally determined FCCs in *Lactococcus lactis* strains.

Enzyme	FCC at wild-type level				Min. fraction of wild-type enzyme level for maximal glycolytic flux	Definite expression level for optimality^∗^	Reference
	
	Growth rate	Glycolytic flux	Lactate flux	Formate flux			
**MG1363**							

LDH	≈0	≈0	≈0	-1.45 ∼-1.27	<59%	No	
TPI	≈0	≈0	≈0	≈-0.25	<40%	No	Solem et al., 2008
GAPDH	≈0	≈0	≈0	≈0	<59%	No	Solem et al., 2003
PFK	≈0	≈0	≈0	≈0	≈100%	Slightly	Andersen et al., 2001b; Koebmann et al., 2005
PYK	≈0	≈0	≈0	0.9 ∼ 1.1	≈100%	Yes	Koebmann et al., 2005

**IL1403**							

TPI	≈0	≈0	≈0	≈0	<75%	No	Solem et al., 2008
PGM	≈0	≈0	≈0	≈0	≈ 100%	Yes	Solem et al., 2010
ENO	≈0	≈0	≈0	≈0	≈100%	Yes	Koebmann et al., 2006


*^∗^‘Definite expression level for optimality’ refers to a unique maximum of growth rate and glycolytic flux at the wild-type enzyme level. LDH, lactate dehydrogenase; TPI, triosephosphate isomerase; GAPDH, glyceraldehyde 3-phosphate dehydrogenase; PFK, phosphofructokinase; PYK, pyruvate kinase; PGM, phosphoglycerate mutase; ENO, enolase*.

**FIGURE 2:**
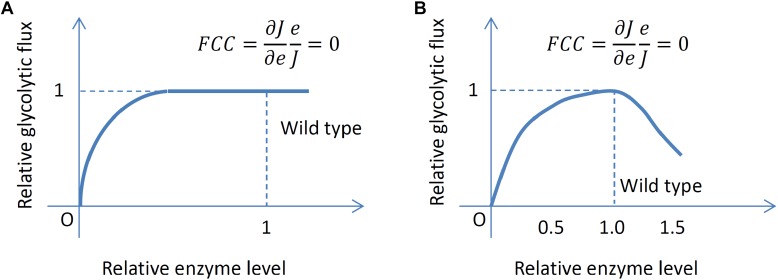
Two different scenarios leading to a zero FCC. **(A)** An enzyme is present in excess and perturbing its level slightly does not change the flux. **(B)** The level of an enzyme allows maximum flux and perturbing the level slightly always decreases the flux.

Interestingly, usually each individual enzyme appears to have no control on growth rate and glycolytic flux at the wild-type enzyme level, including LDH (Andersen et al., 2001a), GAPDH (Solem et al., 2003), PFK, PYK (Koebmann et al., 2005) and triosephosphate isomerase (TPI) (Solem et al., 2008) for MG1363; TPI, enolase (Koebmann et al., 2006) and phosphoglycerate mutase (PGM) (Solem et al., 2010) for IL1403. Among these enzymes, some are present in the wild type in significant excess for attaining maximum glycolytic flux, such as LDH, TPI and GAPDH whereas some enzymes appear to be optimally expressed in the wild type for maximum glycolytic flux, such as PFK, PYK, PGM and enolase (ENO). For the latter set of enzymes, when the expression level is increased or decreased slightly, the growth rate and glycolytic flux decrease. This property of maximum growth rate and glycolytic flux in the wild type also leads to a zero FCC at the wild-type level. Figure 2 illustrates the two different scenarios leading to zero FCCs in the wild type encountered in the experimental studies of glycolytic flux control by glycolytic enzymes. In the literature, nonetheless, the possible consequences and interpretation of these observations have not been discussed thoroughly.

The reason for the zero flux control for these important enzymes also remains elusive. One possible explanation is that glycolysis is already running at its maximum possible rate or the control is distributed over many enzymes (Koebmann et al., 2002a). Another conjecture is that glycolysis is so optimized throughout evolution that the true FCCs cannot be measured due to optimal regulation of protein expressions which somehow counteracts the effect of modulating an enzyme by reallocating the protein expression profile (Teusink et al., 2011). If this is true, the calculated rate is not the defined partial derivative because the concentrations of other enzymes are also functions of the concentration of the perturbed enzyme. The conflicting results on the role of GAPDH from different studies also highlight the difficulty of studying flux control. In the earlier study, nearly full control of glycolytic flux by GAPDH in *L. lactis* Wg2 and NCDO2118 was found (Poolman et al., 1987; Even et al., 1999) but zero control was found in *L. lactis* MG1363 (Solem et al., 2003). One possible explanation is the intrinsic difference between the two strains as the GAPDH level was found to be two-fold higher in MG1363 compared to Wg2. Another possibility is the difference in experimental methods. GAPDH’s activity in Wg2 was only inhibited but not increased whereas both under- and over-expression of GAPDH were included in the study of MG1363. This can lead to contradictory estimation of FCCs.

### Hierarchical Regulation Under Different Growth Conditions

Another approach used to distinguish between hierarchical regulation and metabolic regulation was proposed by Kuile and Westerhoff (2001) by estimating coefficients for the two types of regulation. This approach is to a certain extent working in a reverse sense to the aforementioned approach which estimates the FCC of an enzyme by growing strains with different activities of the enzyme under the same condition and then measuring the changes in activities and fluxes. In contrast, the same strain is cultured under different growth conditions, for example, in chemostat at different dilution rates, or different starvation conditions (Rossell et al., 2006). Then the relative change in enzyme level, measured by enzyme assay, is divided by the relative change in fluxes to define the “hierarchical regulation coefficient” (HRC). It is equal to one in the ideal case of pure hierarchical regulation. The “metabolic regulation coefficient” (MRC) can then be computed by 1 – HRC to account for the change in fluxes not accountable by hierarchical regulation.

In *L. lactis*, several studies have been conducted using this approach. For instance, MG1363 has been grown in chemostat at a dilution rate of 0.1 h^-1^ at different pH, from 4.7 to 6.6 (Even et al., 2003). It was found that when taking the inhibitory effect of pH on enzyme activities into account, metabolic regulation was the dominant force controlling glycolytic flux. Also among the hierarchical regulation, post-transcriptional regulation of gene expression was found to be more prominent than transcriptional regulation by comparing change in mRNA transcript level with change in enzyme activity. MG1363 was grown in chemostat at different dilution rates from 0.15 to 0.6 h^-1^ and transcriptomes, proteomes, enzyme activities were quantified simultaneously (Puri, 2014). Similar conclusions were reached. For dilution rates between 0.15 and 0.5 h^-1^, the changes in flux through most enzymes were predominantly caused by metabolic regulation instead of hierarchical regulation except for alcohol dehydrogenase (ADHE) and possibly pyruvate formate lyase (PFL) whose concentrations decreased as the dilution rate increased and the flux through mixed-acid fermentation pathway decreased. So these two enzymes probably controlled the switch between fermentation modes but not the glycolytic flux. Significant hierarchical regulation only occurred during transition from 0.5 to 0.6 h^-1^ in which the expression of several enzymes were found to have changed, probably due to the effect of CCR by the regulatory protein CcpA. Indeed, similar results of the lack of significant change in expression of glycolytic enzymes have also been observed in an accelerostat study on IL1403 in which the dilution rate increased very slowly from 0.1 to 0.6 h^-1^ to obtain different steady states (Lahtvee et al., 2011). Only PGM was found to show changes in expression.

### Metabolic Regulation

Metabolic regulation is not easy to discover because it usually involves interactions between an enzyme and metabolites other than substrates and products of that enzyme. Extensive *in vitro* enzyme characterization is required to identify possible effector metabolites and experiments for confirmation of *in vivo* regulatory roles can even be more difficult to design. As mentioned above some studies indicated that metabolic regulation was the main driving force for flux regulation and meanwhile many pieces of knowledge on particular regulatory relationships are available, nonetheless, a clear and integrative picture of how different types of metabolic regulation work together to explain most of the known experimental results still remains elusive.

#### Negative Feedback on PTS by FBP and Inorganic Phosphate

One example of the metabolic regulation of glycolysis is the regulation of the phosphorylation of HPr protein (HPr/HPr-Ser-P) by FBP, ATP and inorganic phosphate (Pi) mentioned previously. Since HPr helps sugar uptake through PTS but HPr-Ser-P does not, a high level of FBP due to a high rate of sugar uptake causes more HPr to be phosphorylated into HPr-Ser-P, which eventually slows down the sugar uptake thus forming a negative feedback loop. This loop may help to stabilize the glycolytic flux, especially against sudden changes in sugar availability (Teusink et al., 2011). The question of whether this negative feedback loop poses a bottleneck on maximum glycolytic flux, nevertheless, remains unanswered.

#### Feed-Forward on PYK by FBP

Besides the role in PTS, FBP has also been known to be an activator for PYK (Thompson, 1987). A kinetic study of glycolytic intermediates in glucose-pulse experiments using NMR found that the FBP level rose to a peak during glucose uptake and started to drop after glucose was exhausted and until a certain low FBP level, PEP started to accumulate and remained at a high level during glucose starvation (Voit et al., 2006). The authors proposed that the low FBP level reflecting low supply of glucose could serve as a way to preserve high PEP pool by inhibiting PYK which consumes PEP during sugar starvation for future rapid sugar uptake through PTS. Others, nonetheless, observed that such an activation relationship was also preserved in other organisms including those without PTS and remained conservative about the role of this FBP-PYK relation in glycolysis (Teusink et al., 2011). They suggested another possible role in which a high FBP level could serve as a signal for PYK to remove the phosphoglycerate compounds in favor of a high flux through GAPDH which operated close to thermodynamic equilibrium and was thus sensitive to mass action.

#### Global Cofactors: NADH/NAD^+^ Ratio

Another interesting example is how the glycolytic flux responds to cofactor levels, e.g., NADH/NAD^+^ and ATP/ADP. NADH/NAD^+^ ratio was first proposed by Garrigues et al. (2001) to be an important factor for regulating the glycolytic flux in *L. lactis* NCDO2118 based on findings in a study where the strain was exponentially growing on three sugars, glucose, galactose and lactose, with decreasing glycolytic fluxes. The following observations were made: (i) the *in vitro* activity of GAPDH was almost completely inhibited by a NADH/NAD^+^ ratio higher than 0.05; (ii) the NADH/NAD^+^ ratio positively correlated with the glycolytic flux and was as high as 0.08 on glucose (severe inhibition of GAPDH expected); (iii) high pools of metabolites upstream of GAPDH were found including FBP, GAP and dihydroxyacetone phosphate (DHAP) (suggesting insufficient GAPDH activity to metabolize GAP). A later study by the same group on MG1363 found the same correlation between NADH/NAD^+^ ratio and glycolytic flux, but the factors determining the ratio remained unknown (Garrigues et al., 2001).

#### Global Cofactors: ATP/ADP Ratio

Glycolytic kinetics in non-growing cells of *L. lactis* has been studied using *in vivo* NMR by Neves et al. (1999). The kinetic model built in the study fitted with experimental data predicted that conversion of PEP into pyruvate by PYK is inhibited by a high ATP surplus, i.e., high ATP/ADP ratio, which in turn inhibits NAD^+^ regeneration by LDH and in this way, restricts the glycolytic flux. This somehow provided an explanation for the positive correlation between NADH/NAD+ ratio and glycolytic flux observed in other studies (Garrigues et al., 1997). Later NMR study by the same group focusing on the role of NADH and NAD^+^ found that GAPDH was able to sustain a flux as high as in the wild-type MG1363 in a LDH-knockout strain in which the NADH concentration was 1.5 mM while the inhibitory constant of NADH for GAPDH was found to be 0.4 mM (Neves et al., 2002). The authors, to a certain extent, dismissed the control by GAPDH and NADH, and proposed ATP, ADP and Pi as important regulatory metabolites in glycolysis. When interpreting these results, however, one should bear in mind that that *in vivo* NMR studies often deal with non-growing *L. lactis*, so the results obtained can possibly be different from ones derived using exponentially growing cells.

To test the *in vivo* role of ATP/ADP ratio in growing *L. lactis*, Koebmann et al. (2002b) decreased the intracellular ATP/ADP ratios in MG1363 by expressing ATPase using a synthetic promoter library. Surprisingly, for these strains growing exponentially on glucose, the glycolytic flux showed no significant change over a large range of ATP/ADP ratio from around 9 in the wild type to around 5 in the strain with the highest expression of ATPase. When these strains were in a non-growing state (achieved by resuspending cells in media without amino acids and vitamins), however, the glycolytic flux increased with ATPase activities until a level close to that observed for growing wild type cells. These observations suggest the possibility that glycolysis already operates at its maximal rate in the wild type. Another possible situation suggested by the authors is that although lowering the ATP/ADP ratio might stimulate glycolysis (e.g., by increasing the activities of kinases in the pay-off phase), it might eventually reduce the activity of PFK, which becomes a bottleneck countering the effect, known as the risk of a “turbo design” in which part of the desirable products are invested in the first place as input (Teusink et al., 1998).

#### Other Factors

Other sources of metabolic regulation considered to be important for regulating glycolytic flux include the inhibition of PYK by Pi (Ramos et al., 2004), inhibition of PFK by PEP (Papagianni and Avramidis, 2012) and inhibition of GAPDH by NADH (Garrigues et al., 1997, 2001). Hoefnagel et al. (2002) integrated these three inhibitive relations and the activation of PYK by FBP in a single kinetic model with all rate equations and parameters adopted from literature without fitting. The model succeeded in simulating the observed kinetic behavior during glucose run-out experiments including the rapid increase in PEP and Pi, decrease in ATP and slow depletion of FBP. The authors reasoned the following sequence of kinetic responses upon glucose depletion: (1) Less Pi is retained in G6P, F6P and FBP; (2) Pi increases and inhibits PK; (3) PEP increases due to inhibited PK and no glucose for PTS and thus less pyruvate available; (4) Less substrate for LDH and thus NADH accumulates; (5) GAPDH is inhibited by NADH.

## Redirection of the Glycolytic Flux to Different Biochemicals

**FIGURE 3:**
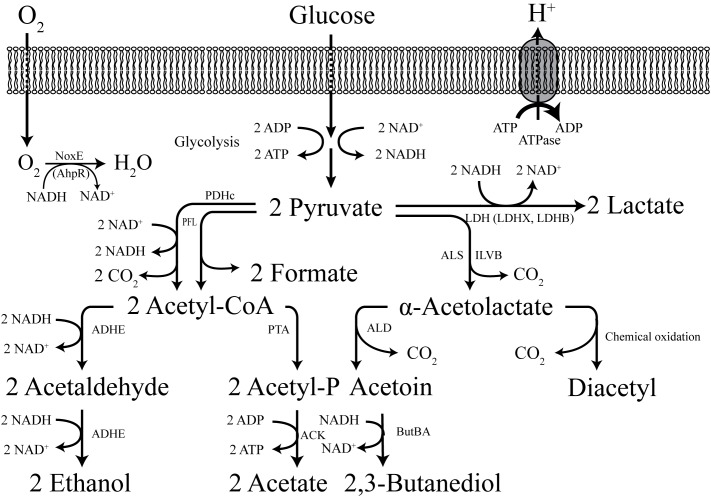
The central metabolism of *L. lactis*. LDH, lactate dehydrogenase; LDHX and LDHB, LDH homologs; ALS (ILVB), α-acetolactate synthase; ALD, a -acetolactate decarboxylase; ButBA, diacetyl reductase and butanediol dehydrogenase; PDHc, pyruvate dehydrogenase complex; PFL, pyruvate formate lyase; PTA, phosphotransacetylase; ADHE, alcohol dehydrogenase; ACK, acetate kinase; Nox, NADH oxidase; ATPase, (F_1_F_0_)-ATPase.

Due to its high glycolytic flux and its safe background, *L. lactis* has been engineered into a cell factory for production of a broad range of interesting biochemicals, from biofuels and food ingredients to vitamins and pharmaceutical precursors (Liu, 2017). Most of these biochemicals are derived from glycolytic precursors. Early efforts on metabolic engineering of *L. lactis* were primarily directed at the manipulation of the pyruvate node. The native metabolic pathways can drive the pyruvate flux to lactate via LDH, or to formate and acetyl-CoA by PFL. Then acetyl-CoA can either be converted into ethanol via the bi-functional ADHE or to acetate via phosphotransacetylase (PTA) and acetate kinase (ACK), subsequently (Figure 3).

### Production of Lactate and Biofuels

#### Lactate

The natural main product of *L. lactis* is L-lactate. *L. lactis* ATCC 19435 has been reported to be able to produce around 100 g/L lactate when using a medium containing whole wheat flour hydrolysate, with a high productivity of 3.0 g/L/h (Hofvendahl and Hahn-Hägerdal, 1997). One of the limiting factors that have prevented the widespread use of *L. lactis* as a cell factory for lactate has been its fastidious nature and a relatively low pH tolerance when compared with various *Lactobacillus* species or yeast (van Hylckama Vlieg et al., 2006; Mazzoli et al., 2014).

Cheaper substrates, e.g., lignocellulose hydrolysates, could serve as a potential feedstock for some *L. lactis* strains that are able to metabolize both the pentoses and hexoses contained, and one research group has investigated the non-dairy strain *L. lactis* IO-1, which is capable of metabolizing xylose (Tanaka et al., 2002). It was demonstrated that xylose could be metabolized via both the phophoketolase pathway and the pentose phosphate pathway into lactate. The flux distribution between the two different pathways was affected by xylose concentration, and a more homolactic profile was attained at high xylose concentrations. Sirisansaneeyakul et al. (2007) used immobilized *L. lactis* IO-1 for producing lactate from glucose and achieved an extremely high volumetric productivity of 4.5 g/L/h using a packed bed of encapsulated cells where the broth was recycled. Another interesting work demonstrated that by expressing the *Escherichia coli* chaperone DnaK in *L. lactis* NZ9000, it was possible to improve multiple-stress tolerance (high temperature, salts, lactate and alcohol) and to increase lactate production (Abdullah-Al-Mahin et al., 2010).

#### Ethanol

The demand for liquid fuels is growing and microbial-based production of biofuels could be an attractive way to increase the supply and decrease the cost. There have been attempts to use *L. lactis* for producing ethanol, but most of them only had limited success. Possible reasons for this could be a too low expression level of heterologous pyruvate decarboxylase (PDC) and alcohol dehydrogenase (AdhB) from *Zymomonas mobilis*. Liu et al. (2005) introduced PDC from *Zymobacter palmae* in *L. lactis* and only observed the accumulation of acetaldehyde. Similarly, Nichols et al. (2003) expressed PDC and AdhB from *Z. mobilis* in *L. lactis* and still failed to drive the flux from lactate fermentation. However, our group several years ago successfully constructed a recombinant *L. lactis*, which was able to produce ethanol as the sole fermentation product (87% of the carbon flux was directed to ethanol production) (Solem et al., 2013). The homo-ethanol producer incorporated the knock-out of several genes encoding the three LDH homologs (*ldh, ldhB*, and *ldhX*), PTA and native ADHE with the introduction of codon-optimized PDC/AdhB from *Z. mobilis* under the synthetic promoter library. Further, this strain was modified by introducing a functional lactose-metabolism. By developing a cheap growth medium containing whey waste and processed corn steep liquor (Liu et al., 2016b), efficient and cheap production of ethanol was accomplished with a titer of 41 g/L ethanol. On the other hand, we also explored the possibility to use the non-dairy *L. lactis* strain KF147 to produce ethanol as the only dominant product from xylose, although the final titer was low. This work demonstrated the great potential of using the more metabolically diverse non-dairy *L. lactis* strains for bio-production from xylose containing feedstocks (Petersen et al., 2017).

#### Butanol Isomers

Butanol has a higher energy density and lower hygroscopicity than ethanol (Peralta-Yahya et al., 2012; Generoso et al., 2015). There have been attempts at engineering *L. lactis* into producing butanol isomers, as they are excellent fuel additives. One advantage of using *L. lactis* as a butanol-producing host is its high butanol tolerance (*L. lactis* can tolerate more than 2% while *E. coli* can only tolerate 1%) (Hviid et al., 2017). Liu et al. (2010) introduced the *Clostridium beijerinckii* P260 thiolase, a key enzyme for re-directing acetyl-CoA to butanol, into *L. lactis*, and enabled production of 28 mg/L butanol, which demonstrates that it is feasible to use *L. lactis* as a production platform. *L. lactis* was also reported to be able to produce isobutanol via the valine degradation pathway (Priyadharshini et al., 2015). In both of these studies the performance of producing strains was clearly insufficient for commercial production, and one reason for the low titer could be a too low availability of acetyl-CoA, the precursor for butanol/isobutanol. In *L. lactis*, acetyl-CoA can be either formed by PFL or by the pyruvate dehydrogenase complex (PDHc) (Figure 3), where PFL is only active in the absence of oxygen and PDHc is quite sensitive to the NADH/NAD^+^ ratio (Snoep et al., 1993). It thus appears that one way to ensure an adequate supply of acetyl-CoA is to introduce a robust PDHc. One of the native enzymes in the isobutanol pathway of *L. lactis* is KDC (α-ketoisovalerate decarboxylase), and this enzyme has been widely used in different strain platforms for efficient isobutanol production (Atsumi et al., 2008).

#### 2,3-Butanediol Isomers

2,3-butanediol (2,3-BDO) is also considered to be an excellent biofuel as well as a good platform-chemical with many applications, e.g., for making plastics, perfumes and pharmaceuticals (Ji et al., 2011). Crow (1990) characterized the two native 2,3-butanediol dehydrogenases from *L. lactis* and identified that one generates *meso*-2,3-BDO from acetoin whereas the other forms an optical isomer generated from diacetyl. Gaspar et al. (2011) overexpressed the native α-acetolactate synthase (Als) and acetoin reductase (ButA) in an LDH-deficient strain, and demonstrated that 67% of the glucose flux could be redirected to 2,3-BDO concurrently with the production of formate (0.65 mol/mol glucose) and ethanol (0.59 mol/mol glucose) anaerobically. Recently we constructed several recombinant *L. lactis* strains for high-titer and high-yield production of 2,3-BDO isomers: *meso*-2,3-BDO, (*R*,*R*)-2,3-BDO and (*S*,*S*)-2,3-BDO (Kandasamy et al., 2016; Liu et al., 2016a). We introduced EcBDH (butanediol dehydrogenase from *Enterobacter cloacae*) into our platform strain *L. lactis* where deletions had been introduced into the genes encoding LDH, PTA, ADHE and ButBA, then further introduced the capacity to metabolize lactose, which enabled high-titer (51 g/L) and high-yield (0.47 g/g lactose) production of *meso*-2,3-BDO from whey permeate (dairy byproduct). Similarly, we demonstrated that the alcohol dehydrogenase SadB from *Achromobacter xylosooxidants* was able to produce (*R*,*R*)-2,3-BDO with a titer of 32 g/L and a yield of 0.40 g/g lactose. Since the production pathway of *meso*-2,3-BDO and (*R*,*R*)-2,3-BDO requires one NADH per mole glucose, the excess of NADH is consumed by controlling the Nox activities through limiting oxygen levels. It is not feasible or very difficult to control oxygen levels in large scales, and for this reason we developed a robust strategy to facilitate chemicals production by fine-tunning the respiration capacity (Liu et al., 2017). The efficient production of *meso*-2,3-BDO and (*R*,*R*)-2,3-BDO demonstrated the great potential of *L. lactis* to become an efficient cell factory for synthesis of biochemicals. Regarding the synthesis of (*S*,*S*)-2,3-BDO, which is another optical isomer of 2,3-BDO, we developed a special strategy, which is to combine enzymatic reactions (glycolysis and from diacetyl to (*S*,*S*)-2,3-BDO) and a non-enzymatic reaction (from α-acetolactate to diacetyl). By using a metabolically engineered *L. lactis* strain a titer of 6.7 g/L (*S*,*S*)-2,3-BDO was achieved from glucose (Liu et al., 2016a). To the best of our knowledge, this is the first time this chemical has been produced by direct microbial fermentation. The key to the success was to use a combination of a biocompatible catalyst, for speeding up the conversion of α-acetolactate into diacetyl, and a robust diacetyl reductase from *E. cloacae*.

### Production of Food Ingredients and Vitamins

#### Diacetyl

The long record of safe use of *L*. *lactis* for food fermentations makes *L. lactis* an excellent choice as a host for producing food ingredients. There have been many attempts to use this organism for producing diacetyl, which is a potent flavor compound that contributes to the buttery aroma of many fermented foods, such as cheese, butter and butter milk. *L. lactis* subsp. *lactis* bv. diacetylactis is a native producer of diacetyl, and is widely used in the food industry. Normally the diacetyl-forming ability of this strain is associated with citrate metabolism, where citrate is converted into pyruvate which boosts the pyruvate pool enabling α-acetolactate formation via α-acetolactate synthase (Hugenholtz and Starrenburg, 1992). Since the availability of citrate is very low in the food raw materials, there have been efforts to change the native metabolism of *L. lactis*, either by random mutagenesis or by rational design. Monnet et al. (2000) selected mutants of *L. lactis* subsp. *lactis* bv. diacetylactis that were deficient in α-acetolactate decarboxylase (ALD) and had an overall low LDH activity, and demonstrated formation of 6 mM diacetyl, 30 mM acetoin and 12 mM α-acetolactate when the strains were grown in milk supplemented with catalase under aerobic conditions. Hugenholtz et al. (2000) combined NADH-oxidase (NoxE) overexpression and ALD inactivation, and achieved 1.6 mM of diacetyl using resting cells under aerobic conditions, which corresponded to a conversion efficiency of 16% (57% α-acetolactate, 21% acetate). Guo et al. (2012) constructed a promoter library for driving the expression of NoxE and could achieve a slightly higher diacetyl concentration of 4.16 mM. Recently we developed a novel strategy in *L. lactis*, relying on a combination of metabolic engineering, respiration technology and metal-ion catalysis, which turned out to be successful. The homolactic *L. lactis* was converted into a homo-diacetyl producer with a very high titer (95 mM) and a high yield (87% of the theoretical maximum) (Liu et al., 2016a).

#### Acetoin Isomers

Another important flavor compound, acetoin ((3*R*)-acetoin) can be produced through the native pathway in *L. lactis* from pyruvate by the subsequent Als and ALD. It was reported that *L*. *lactis* subsp. *lactis* bv. diacetylactis could produce 5.4 mM acetoin under fully aerated conditions (100% oxygen saturation) after the citrate had been completely consumed (Bassit et al., 1993). We recently achieved high-level production of acetoin (306 mM, 27 g/L) using metabolically engineered *L. lactis* from whey permeate (Kandasamy et al., 2016; Liu et al., 2016c). The high titer is mainly due to the completely rerouted metabolism.

We also managed to produce another isomer of acetoin, (3*S*)-acetoin. It can also serve as a flavor compound, but has other applications as well, e.g., for the synthesis of novel optically active α-hydroxyketone derivatives, pharmaceutical precursors and liquid crystal composites (Xiao and Lu, 2014). (3*S*)-acetoin can be produced from diacetyl with the aid of diacetyl reductase (DAR). By manipulating cofactor availability and using metal ion catalysis for speeding up non-enzymatic oxidative decarboxylation of α-acetolactate, (3*S*)-acetoin can be produced at 66 mM (71% of the theoretical maximum) (Liu et al., 2016d). To the best of our knowledge this is the first time this isomer has been made by microbial fermentation.

#### Alanine

Hols et al. (1999) successfully rerouted the carbon flux of *L. lactis* toward the production of alanine, which is a natural sweetener used as a food ingredient and as a pharmaceutical precursor. The engineered strain was deficient in LDH activity and equipped with alanine dehydrogenase (AlaDH) from *Bacillus sphaericus*. Using resting cells as biocatalyst, alanine (around 200 mM) was the only product formed after optimizing pH and ammonium concentration. Another AlaDH from *Bacillus subtilis* (natto) was demonstrated to have a potential for improving alanine levels in *L. lactis* NZ9000 fermentation broth (Ye et al., 2010).

#### Acetaldehyde

Acetaldehyde is considered as the most important aroma compound in yogurt. In order to achieve formation of more acetaldehyde in dairy products, Bongers et al. (2005) overexpressed PDC from *Z. mobilis* and NoxE in the wild type of *L. lactis*. They found it was possible to redirect 50% of the carbon flux in resting cells to acetaldehyde, which accumulated to 9.5 mM. From this work it was clear that PDC with its low Km for pyruvate (0.3 mM) could efficiently drain the pyruvate pool, whereas this is more difficult to achieve using Als that has a very high Km for pyruvate (50 mM) (Zullian et al., 2014).

#### Vitamins – Riboflavin

**FIGURE 4:**
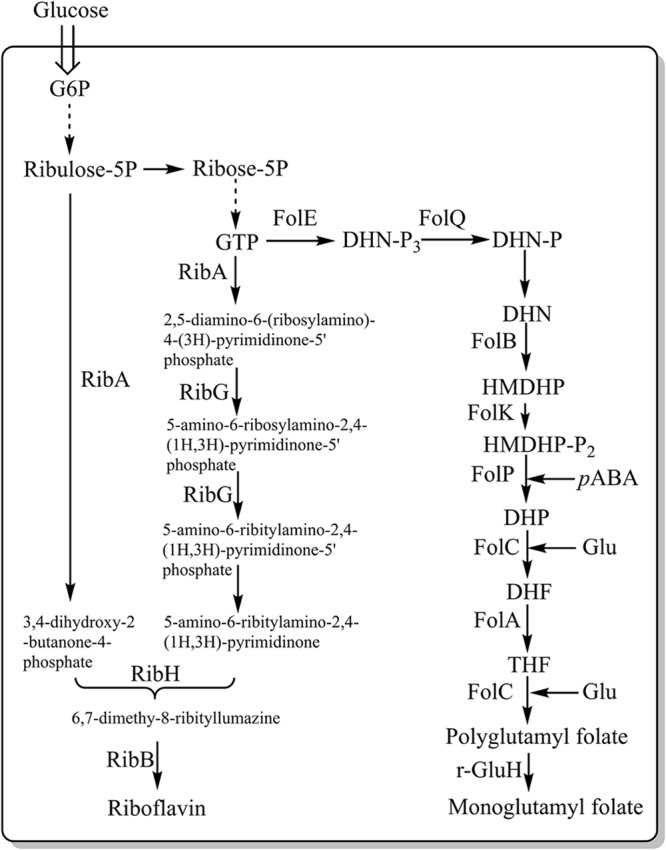
Metabolic pathways involved in the synthesis of riboflavin and folate in LAB. FolE, GTP cyclohydrolase I; FolQ, dihydroneopterin triphosphate pyrophosphohydrolase; FolB, dihydroneopterin aldolase; FolK, hydroxymethyldihydropterin pyrophosphokinase; FolP, dihydropteroate synthase; FolC, dihydrofolate synthase; FolA, dihydrofolate reductase; PabAB, chorismate synthetase component I and II; PabC, 4-amino-4-deoxychorismate lyase; Glu, glutamate; pABA, *para*-aminobenzoic acid; γ-GluH, gamma-glutamyl hydrolase; RibA, GTP cyclohydrolase II/3,4-dihydroxy-2-butanone-4-phosphate synthase; RibG, riboflavin-specific deaminase/reductase; RibH, riboflavin synthase (beta subunit); and RibB, riboflavin synthase (alpha subunit). Dotted arrows represent multiple consecutive steps in the pathways.

Vitamins are vital nutrients needed for the normal functioning of living organisms. Vitamin deficiencies occur commonly, and are often associated with certain health problems, however, deficiencies can be overcome by supplementation, e.g., by fortifying foods with vitamins. Several studies have focused on engineering *L. lactis* into synthesizing B vitamins, such as riboflavin (vitamin B2) and folate (vitamin B11) (Thakur et al., 2016). Burgess et al. (2004) overexpressed the rib operon (*ribGBAH*) (Figure 4) and achieved high level production of riboflavin (24 mg/L). Furthermore, they developed a strategy to select and isolate spontaneous riboflavin-overproducing *L. lactis*, which was to use the toxic riboflavin analog roseoflavin. Several mutants exhibited a significant higher-level of riboflavin (around 900 mg/L) (Burgess et al., 2004). Recently our group selected a riboflavin overproducer through the combination of roseoflavin resistance, random mutagenesis and microfluidic screening. The mutant could increase the riboflavin content in milk to 2.81 mg/L whereas the wild-type reduced the riboflavin content of milk to 0.66 mg/L (Chen J. et al., 2017). These results indicate a great potential for *L. lactis* to become an efficient producer of riboflavin, either by using GMO or non-GMO approaches. LeBlanc et al. (2005) carried out an animal test and found that feeding rats with live *L. lactis* cells overproducing riboflavin could stimulate their growth.

#### Vitamins – Folate

*Lactococcus lactis* is a good producer of folate, predominantly in the form of polyglutamyl. The Fol operon, which includes *folA, folB, folKE, folP* and *folC*, was found to be involved in folate biosynthesis (Figure 4). The overexpression of *folKE* in *L. lactis* resulted in an increase in extracellular folate production by 8-fold (from 10 to 80 ng/ml), while the total folate production increased by 2-fold (from 100 to 180 ng/ml) (Sybesma et al., 2003). Wegkamp et al. (2007) further documented that the overexpression of the folate operon and para-aminobenzoic acid (pABA) gene clusters, where pABA is the important precursor for folate synthesis, resulted in production of 2.7 mg/L folate per optical density unit at 600 nm, which is 80 times higher than what the wild type is capable of.

### Production of Polysaccharides and Plant Metabolites

#### Exopolysaccharides and Hyaluronic Acid

Exopolysaccharides (EPSs) are long-chain polysaccharides that are secreted into their surroundings during bacterial growth. EPSs contribute to the texture of many dairy products (Laws et al., 2001). It has also been reported that they can function as prebiotics, cholesterol lowering nutraceuticals or immunomodulants (Nwodo et al., 2012). The biosynthesis of EPSs is complex and normally involves a large number of gene products. In *L. lactis*, the genes responsible for EPSs synthesis are located in a large operon containing 14 genes, *epsRXABCDEFGHIJKL*, which is found on a plasmid (van Kranenburg et al., 1997). van Kranenburg et al. (1999) demonstrated that by over-expressing *edsD*, encoding the priming glycosyltransferase, could increase the EPSs production from 113 to 133 mg/L. Further through increasing the expression level of the entire *eps* gene cluster, EPSs production was increased further to 343 mg/L (Boels et al., 2003a,b). However, an increase in the concentration of sugar-phosphates (Glu-6P, Glu-1P) or sugar nucleotides (UDP-glucose, UDP-galactose) did not affect production of EPSs significantly. Recently the EPSs biosynthetic pathways were re-engineered to enable production of hyaluronic acid (HA), which is another kind of polysaccharide with various applications in pharmaceuticals and foods (Shah et al., 2013). HA is synthesized from the polymerization of UDP-glucuronic acid and UDP-N-acetylglucosamine, two precursors of cell-wall components, through HA synthase. The recombinant expression of HA synthase from *Streptococcus equi* subsp. *zooepidemicus* in *L. lactis* resulted in 0.08 g/L HA, and the coexpression of HA synthase and uridine diphosphate-glucose dehydrogenase (UDP-GlcDH) significantly enhanced the HA production to 0.65 g/L (Chien and Lee, 2007). Prasad et al. (2010) coexpressed UDP-glucose pyrophosphorylase as well as HA synthase and UDP-GluDH and achieved 1.8 g/L HA in bioreactor experiment with controlled pH and aeration. These promising results demonstrate the great potential of *L. lactis* as a good platform for the production of functional polysaccharides.

#### Plant Metabolites

In the last 10 years, *L. lactis* has been studied as a host for the expression of plant derived genes to produce plant metabolites. Song et al. (2012) expressed b-sesquiphellandrene synthase from *Persicaria minor* in *L. lactis* successfully and confirmed the production of b-sesquiphellandrene, which is a valuable sesquiterpene and has excellent antimicrobial and antioxidative properties. The co-expression of 3-hydroxy-3-methyglutaryl coenzyme A reductase, which is the limiting enzyme in the mevalonate pathway, increased the b-sesquiphellandrene level to 109 nM (Song et al., 2012). Hernández et al. (2007) cloned two genes from strawberry, encoding an alcohol acyltransferase and a linalool/nerolidol synthase and expressed them in *L. lactis*, which resulted in the production of octyl acetate (1.9 mM) and linalool (85 nM). The production of plant metabolites in *L. lactis* is just in the beginning phase and these previous results demonstrate a great potential.

## Concluding Remarks and Future Prospects

In many single strain omics and systems biology studies, cells are scrutinized under steady-state conditions, which is indeed useful for the purpose of exploring and understanding the regulation of cellular metabolism of LAB, e.g., glycolysis. A large number of studies have also demonstrated how helpful this knowledge and understanding is for the design and engineering of robust LAB cell factories for producing different high-value chemicals. Systems biology methods including omics analysis also have allowed us to understand how LAB respond to environmental changes. In the future, other variables should be considered for the study of LAB communities e.g., temporal and spatial change of community composition due to biotic/abiotic factors in the production process, which significantly increase complexity of the research. These changes have showed great influences on the cellular states of the microbial community. So, in the future, the improved meta-omics analysis resolution, and use of properly simplified models are some of the keys for the systems biology study of the LAB community, which will benefit the understanding of these organisms in the real-life applications.

## Author Contributions

All authors listed have made a substantial, direct and intellectual contribution to the work, and approved it for publication.

## Conflict of Interest Statement

The authors declare that the research was conducted in the absence of any commercial or financial relationships that could be construed as a potential conflict of interest.

## Abbreviations

γ-GluH: gamma-glutamyl hydrolase
ACK: acetate kinase
ADHE: alcohol dehydrogenase
ALD: α-acetolactate decarboxylase
ALE: adaptive laboratory evolution
ALS (ILVB): α-acetolactate synthase
ATPase: (F_1_F_0_)-ATPase
ButBA: diacetyl reductase and butanediol dehydrogenase
FBA: flux balance analysis
FolA: dihydrofolate reductase
FolB: dihydroneopterin aldolase
FolC: dihydrofolate synthase
FolE: GTP cyclohydrolase I
FolK: hydroxymethyldihydropterin pyrophosphokinase
FolP: dihydropteroate synthase
FolQ: dihydroneopterin triphosphate pyrophosphohydrolase
Glu: glutamate
GMO: genetically modified organism
LAB: lactic acid bacteria
LDH: lactate dehydrogenase
Nox: NADH oxidase
pABA: *para*-aminobenzoic acid
PabAB: chorismate synthetase component I and II
PabC: 4-amino-4-deoxychorismate lyase
PDHc: pyruvate dehydrogenase complex
PFL: pyruvate formate lyase
PTA: phosphotransacetylase
PTS: phosphotransferase system
RibA: GTP cyclohydrolase II/3,4-dihydroxy-2-butanone-4-phosphate synthase
RibB: riboflavin synthase (a subunit)
RibG: riboflavin-specific deaminase/reductase
RibH: riboflavin synthase (b subunit)
SNV: single nucleotide variation
